# Erythropoietin, Forkhead Proteins, and Oxidative Injury: Biomarkers and Biology

**DOI:** 10.1100/tsw.2009.121

**Published:** 2009-10-02

**Authors:** Kenneth Maiese, Jinling Hou, Zhao Zhong Chong, Yan Chen Shang

**Affiliations:** ^1^ Division of Cellular and Molecular Cerebral Ischemia, Wayne State University School of Medicine, Detroit, MI, USA; ^2^ Department of Neurology and Anatomy and Cell Biology, Wayne State University School of Medicine, Detroit, MI, USA; ^3^ Barbara Ann Karmanos Cancer Institute, Wayne State University, School of Medicine, Detroit, MI, USA; ^4^ Center for Molecular Medicine and Genetics, Wayne State University School of Medicine, Detroit, MI, USA; ^5^ Institute of Environmental Health Sciences, Wayne State University School of Medicine, Detroit, MI, USA

**Keywords:** aging, Alzheimer’s disease, angiogenesis, apoptosis, cancer, cardiac, diabetes, erythropoietin, forkhead transcription factors, immune system, ischemia, neurodegeneration, oxidative stress, sirtuins, stem cells, vascular disease, Wnt, wingless

## Abstract

Oxidative stress significantly impacts multiple cellular pathways that can lead to the initiation and progression of varied disorders throughout the body. It therefore becomes imperative to elucidate the components and function of novel therapeutic strategies against oxidative stress to further clinical diagnosis and care. In particular, both the growth factor and cytokine erythropoietin (EPO), and members of the mammalian forkhead transcription factors of the O class (FoxOs), may offer the greatest promise for new treatment regimens, since these agents and the cellular pathways they oversee cover a range of critical functions that directly influence progenitor cell development, cell survival and degeneration, metabolism, immune function, and cancer cell invasion. Furthermore, both EPO and FoxOs function not only as therapeutic targets, but also as biomarkers of disease onset and progression, since their cellular pathways are closely linked and overlap with several unique signal transduction pathways. Yet, EPO and FoxOs may sometimes have unexpected and undesirable effects that can raise caution for these agents and warrant further investigations. Here we present the exciting as well as the complex role that EPO and FoxOs possess to uncover the benefits as well as the risks of these agents for cell biology and clinical care in processes that range from stem cell development to uncontrolled cellular proliferation.

## INTRODUCTION

### Oxidative Stress and Apoptotic Injury

The generation of reactive oxygen species (ROS) that consist of oxygen free radicals and other chemical entities can result in the development of oxidative stress. Oxygen free radicals can be generated in elevated quantities during the reduction of oxygen and lead to cell injury. ROS can involve superoxide free radicals, hydrogen peroxide, singlet oxygen, nitric oxide (NO), and peroxynitrite[1,2,3]. Most species are produced at low levels during normal physiological conditions and are scavenged by endogenous antioxidant systems that include superoxide dismutase (SOD), glutathione peroxidase, catalase, and small molecule substances such as vitamins C and E. Other closely linked pathways to oxidative stress may be tempered by different vitamins, such as vitamin $D_3$[4] and the amide form of niacin or vitamin $B_3$, nicotinamide[5,6,7,8,9,10,11].

Initial investigations into oxidative stress may have begun with studies that examined the rate of oxygen consumption in organisms. Work by Pearl proposed that increased exposure to oxygen through a high metabolic rate could lead to a shortened life span[12]. Additional work by other investigators demonstrated that increased metabolic rates could be detrimental to animals in an elevated oxygen environment[13]. Current studies show that oxygen free radicals and mitochondrial DNA mutations have become associated with cellular injury, aging mechanisms, and accumulated toxicity for an organism[14].

Oxidative stress leads to the destruction of multiple cell types through apoptotic pathways[15,16,17] and also through autophagy[18]. However, it has also recently been shown that genes involved in the apoptotic process are replicated early during processes that involve cell replication and transcription, suggesting a much broader role for these genes than originally anticipated[19]. Apoptotic-induced oxidative stress in conjunction with processes of mitochondrial dysfunction[20,21,22] can contribute to a variety of disease states, such as diabetes, ischemia, cognitive loss, Alzheimer’s disease[1,23,24], Parkinson’s disease[1,25], Huntington’s disease[1,26], and trauma [1,23,27,28,29]. Oxidative stress can lead to apoptosis in neurons, endothelial cells (ECs), cardiomyocytes, and smooth muscle cells that involve separate as well as overlapping pathways[27,30,31,32,33,34].

Apoptosis is a dynamic process that consists of both the early exposure of membrane phosphatidylserine (PS) residues and the late destruction of genomic DNA[35,36]. Externalization of membrane PS residues is an early event during cell apoptosis[37,38] and can become a signal for the phagocytosis of cells that is controlled by caspase 1 and caspase 3[17,39,40]. The loss of membrane phospholipid asymmetry leads to the exposure of membrane PS residues on the cell surface and assists microglia to target cells for phagocytosis[11,32,41,42,43]. This process occurs with the expression of the phosphatidylserine receptor (PSR) on microglia during oxidative stress[44,45]. It has been shown that blockade of PSR function in microglia prevents the activation of microglia[42,46]. Externalization of membrane PS residues occurs in neurons, vascular cells, and inflammatory microglia during reduced oxygen exposure[17,47,48], β-amyloid (Aβ) exposure[49,50], NO exposure[51,52,53,54,55], and during the administration of agents that induce the production of ROS, such as 6-hydroxydopamine[56]. Membrane PS externalization on platelets also has been associated with clot formation in the vascular system[57].

The cleavage of genomic DNA into fragments[47,58,59] usually occurs after membrane PS exposure[60] and is considered to be a later event during apoptotic injury[32,59,61,62]. Several enzymes responsible for DNA degradation include the acidic cation-independent endonuclease (DNase II), cyclophilins, and the 97-kDa magnesium-dependent endonuclease[1,63]. Three separate endonuclease activities have also been found in neurons that include a constitutive acidic cation-independent endonuclease, a constitutive calcium/magnesium-dependent endonuclease, and an inducible magnesium-dependent endonuclease[64,65].

During oxidative stress, mitochondrial membrane transition pore permeability is also increased[10,32,66,67], a significant loss of mitochondrial $NAD^+$ stores occurs, and further generation of superoxide radicals leads to cell injury[11,68]. Mitochondria are a significant source of superoxide radicals that are associated with oxidative stress[1,69]. Blockade of the electron transfer chain at the flavin mononucleotide group of complex I or at the ubiquinone site of complex III results in the active generation of free radicals, which can impair mitochondrial electron transport and enhance free radical production[44,63]. Furthermore, mutations in the mitochondrial genome have been associated with the potential development of a host of disorders, such as hypertension, hypercholesterolemia, and hypomagnesemia[70,71]. ROS may also lead to cellular acidosis and subsequent mitochondrial failure[23]. Disorders, such as hypoxia[72], diabetes[73,74], and excessive free radical production[65,75,76], can result in the disturbance of intracellular pH.

### Biomarkers in Health and Disease

For biological systems, a "biomarker" can consist of any entity that occurs in the body and that can be measured to predict the diagnosis, onset, or progression of a disease process. A biomarker does not have to be confined to a single entity. As a result, the definition of a biomarker is intentionally broad and application of biomarkers can be used for the determination of specific genes, proteins, products of cellular and biological processes, as well as the response of cells or tissues to therapeutic strategies[77].

Interestingly, some biomarkers can offer the additional benefit to function as a surrogate marker to be able to be used to predict clinical outcome in some cases. For example, biomarkers such as estrogen levels may predict the onset of postmenopausal breast cancer and a poor clinical outcome. In other scenarios, biomarkers may suggest the body's attempt to initiate reparative processes. Novel pathways that involve the cytokine and growth factor erythropoietin (EPO) may indicate that the increased presence of this agent during periods of oxidative stress may lead to cellular mechanisms to protect against ROS[78,79,80]. Furthermore, the activation of transcription factors during tumor invasion that control cell cycle regulation, such as of the forkhead family of the "O" class, may suggest the initiation of cell pathways that are attempting to restrict neoplastic growth[81,82,83]. However, reliance on any single biomarker may be imperfect and lead to initially unpredicted outcomes, such as uncontrolled hypertension or cancer with EPO[78,79,84], or the onset of detrimental apoptotic programs with forkhead transcription factors[36]. A number of other pathways that occur in combination with a particular biomarker during oxidative stress may also influence outcome. In the case of breast cancer, studies suggest that the release of androgens, cytokines, or even changes in body mass and exercise can influence outcome as well as alter the predictability of a specific biomarker[85,86]. For these reasons, it becomes imperative to elucidate the components and function of the novel pathways for EPO and forkhead transcription factors during oxidative stress in order to understand their role not only as biomarkers, but also as therapeutic strategies to offer new insight for clinical care for a number of disease entities.

## THE GROWTH FACTOR AND CYTOKINE ERYTHROPOIETIN (EPO)

### Historical Perspective for EPO

EPO was initially known as "hemopoietine", which could stimulate new red blood cell development. In 1906, Carnot and Deflandre demonstrated that plasma removed from rabbits following a bleeding stimulus that was later injected into control, untreated rabbits would lead to the development of immature red blood cells[78,79,87,88]. A number of other investigators followed these studies and found similar results demonstrating that plasma from bled animals would yield a significant reticulocytosis[89,90,91]. More elegant experiments eventually demonstrated that a rise in hemoglobin levels with reticulocytosis occurred in parabiotic rats when only one partner was exposed to hypoxia, illustrating that depressed oxygen tensions could stimulate EPO production[92]. Later, human EPO protein was purified, which led the way for the cloning of the EPO gene and the development of recombinant EPO for clinical use[93,94].

### Structure and Chemical Properties for EPO

The EPO gene is located on chromosome 7, exists as a single copy in a 5.4-kb region of the genomic DNA, and encodes a polypeptide chain containing 193 amino acids. During the production and secretion of EPO, a 166-amino-acid peptide is initially generated following the cleavage of a 27-amino-acid hydrophobic secretory leader at the amino-terminal. In addition, a carboxy-terminal arginine in position 166 is removed both in the mature human and recombinant human EPO (rhEPO), resulting in a circulatory mature protein of 165 amino acids[80,95]. Once a mature protein, EPO becomes a 30.4-kDa glycoprotein with approximately half of its molecular weight derived from carbohydrates that can vary among species[80]. EPO contains four glycosylated chains, including three *N*-linked and one *O*-linked acidic oligosaccharide side chains. The glycosylated chains are important for the biological activity of EPO and can protect EPO from oxygen radical degradation. EPO is stabilized by the carbohydrate chains[96]. The oligosaccharides in EPO may also protect the protein from oxygen radical activity[97]. The N-glycosylated chains are believed to contribute to the thermal stability of EPO[98]. In addition, the *N*- and *O*-linked chains may be necessary for the production and secretion of the mature EPO[99]. The presence of the carbohydrates is also important in the control of the metabolism of EPO, since EPO molecules with high sialic acid content can be easily cleared by the body through specific binding in the liver[100]. In addition, the biological activity of EPO also relies on two disulfide bonds formed between cysteines at positions 7 and 160, and at positions 29 and 33[95].

## Expression and Regulation of EPO and the EPO Receptor

The principal organs of EPO production and secretion are the kidney, liver, brain, and uterus. EPO production and secretion occurs foremost in the kidney[101]. The kidney peritubular interstitial cells are responsible for the production and secretion of EPO[88]. With the use of cDNA probes derived from the EPO gene, peritubular ECs, tubular epithelial cells, and nephron segments in the kidney have also been demonstrated to be vital cells for the production and secretion of EPO[102,103]. During periods of acute renal failure, EPO may provide assistance for the protection of the kidneys and nephrons[104,105,106]. Other sites of EPO production and secretion occur in the liver and the uterus[107]. Hepatocytes, hepatoma cells, and Kupffer cells of the liver can produce EPO and, in turn, EPO may protect these cells from injury and assist with regeneration[108,109]. In regards to the uterine production of EPO, it is believed that the occurrence of neonatal anemia that can take place in the early weeks after birth may partly result from the loss of EPO production and secretion by placenta[110]. In addition, increased levels of EPO in the fetal plasma and amniotic fluid during gestation may function as a biomarker of intrauertine hypoxia[111].

Although EPO is approved by the U.S. Food and Drug Administration for the treatment of anemia, recent studies demonstrated that EPO is not only required for erythropoiesis, but also functions in other organs and tissues, such as the brain, heart, and vascular system[46,112,113,114,115,116]. EPO production is believed to occur throughout the body[3,80,117] and can be detected in the breath of healthy individuals[118]. In addition, it has been suggested that EPO may provide developmental cognitive support. In experimental animal models, EPO may reduce apoptotic pathways during periods of hyperoxia in the developing brain[119,120]. Furthermore, clinical disorders may have periods of hyperoxia followed by cerebral hypoperfusion and hypoxia that can lead to cerebral injury with associated oxidative stress[121]. In these circumstances, EPO may also be protective, since it can promote neurite outgrowth[122] and may also regulate hemoglobin levels that have recently been associated with cognitive decline[123]. In other work, elevated EPO concentrations during infant maturation were correlated with increased Mental Development Index scores[124] and EPO may prevent the toxic effects of agents used to control cognitive function, such as haloperidol[125].

However, new knowledge that EPO and its receptor are present in the nervous and vascular systems has generated great enthusiasm for the potential clinical applications of EPO, such as in Alzheimer’s disease, Parkinson’s disease[126], cardiac insufficiency[127,128,129,130], cardiac transplantation[131,132], and during coronary artery bypass surgery to prevent renal injury[133]. In the nervous system, primary sites of EPO production and secretion are in the hippocampus, internal capsule, cortex, midbrain, cerebral ECs, and astrocytes[80,95,134,135]. Further work has revealed several other organs as secretory tissues for EPO that include peripheral ECs[136], myoblasts[137], insulin-producing cells[138], and cardiac tissue[80,101].

EPO controls erythroid cell proliferation, differentiation, and survival through its binding to a target cell surface receptor, the EPO receptor (EPOR)[139] (Fig. 1). The EPOR is also expressed in numerous nonerythroid blood lines that include neurons, microglia, astrocytes, and in cerebral ECs[80,95,101,135,136], as well as on myelin sheaths of radicular nerves in human peripheral nerves[140], suggesting both a developmental and potential protective role for EPO in the central and peripheral nervous systems. During gestation, EPO production is increased, but later becomes suppressed following birth to be regulated by the tissue oxygen supply[141]. The EPOR is also expressed in primary cerebral ECs[67,80] as well as in human umbilical veins, bovine adrenal capillaries, and rat brain capillaries[136,142].

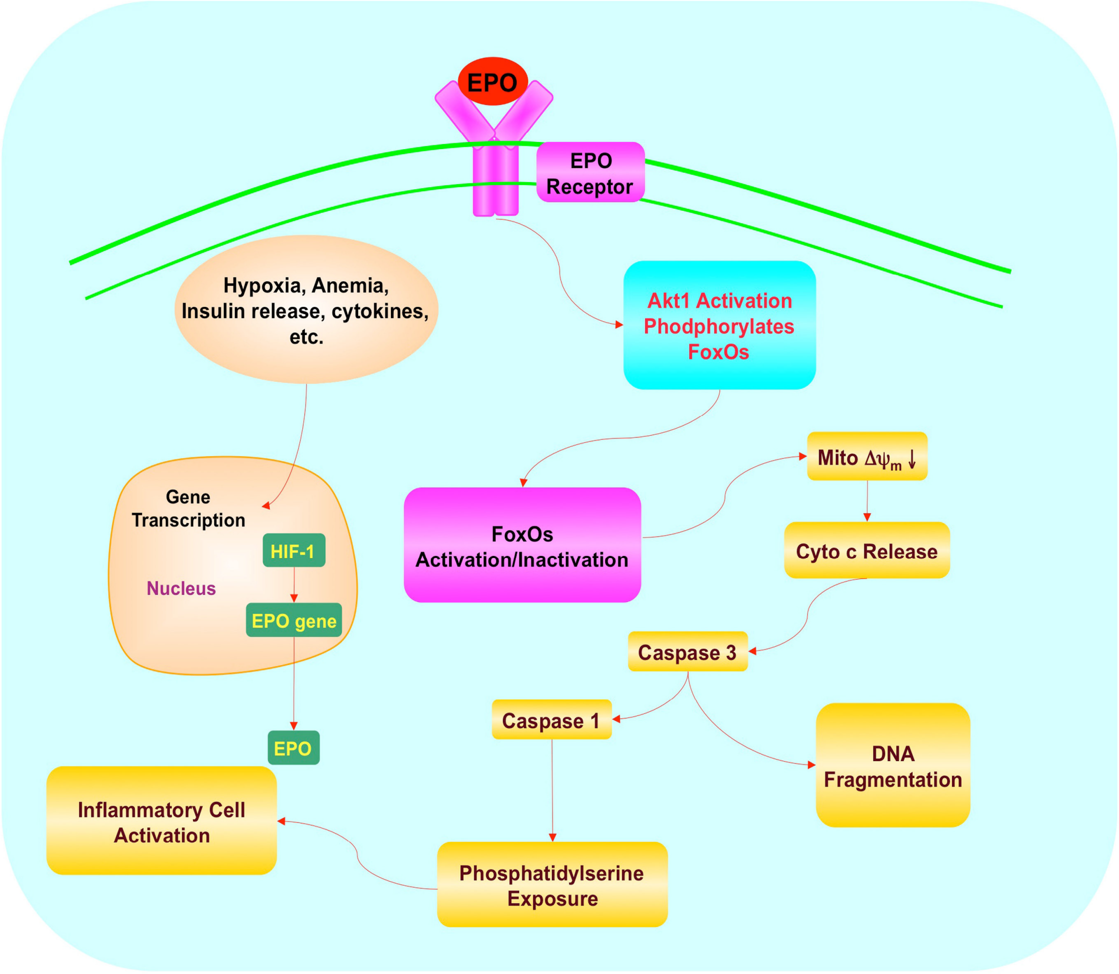

**FIGURE 1.** EPO and forkhead transcription factors (FoxOs) control cell survival and inflammatory cell activation. EPO through the EPOR and FoxOs can modulate cell survival and control inflammatory cell activation through pathways that involve hypoxia-inducible factor 1 (HIF-1) activation (occurs through several stimuli, such as hypoxia, anemia, insulin release, and cytokine exposure), gene transcription, protein kinase B (Akt), mitochondrial membrane potential ($\Delta\Psi_m$), cytochrome c (Cyto-c), and caspases. These pathways can then regulate the onset of early apoptotic injury with PS exposure, late injury with nuclear DNA degradation, and inflammatory cell activation.

Despite the fact that EPO is a critical modulator of erythropoiesis, the presence of a diminished oxygen tension is required, rather than a low concentration of red blood cells[3,78,79,143]. In most tissues, including the brain, hypoxia-dependent expression of EPO and the EPOR are controlled by hypoxia-inducible factor 1 (HIF-1). HIF-1 is essential for the production and secretion of EPO in response to hypoxia. At the transcriptional level, the hypoxia-dependent gene transcription of EPO and the EPOR directly results from the activation of the HIF-1 pathway under hypoxic conditions. Gene transcription of EPO is mediated by the transcription enhancer located in the 3'-flanking region of the EPO gene that specifically binds to HIF-1[80,95]. Yet, hypoxia is not the only condition that can alter the expression of EPO and the EPOR. The production and secretion of EPO in female reproductive organs is estrogen dependent. During the cyclic development of the uterine endometrium, 17β-estradiol can lead to a rapid and transient increase in EPO mRNA in the uterus[144], oviducts, and ovaries[145]. Hypoxic-induced EPO mRNA expression in uterine tissue occurs only in the presence of 17β-estradiol. EPO mRNA expression by hypoxia in the uterus is less pronounced than the EPO expression that occurs in the kidney and the brain[146]. Interestingly, a variety of cellular disturbances may lead to either increased or decreased EPO expression through the control of HIF, such as hypoglycemia, cadmium exposure, raised intracellular calcium, or intense neuronal depolarizations generated by mitochondrial ROS[135,141,147]. Anemic stress, insulin release, and several cytokines, including insulin-like growth factor, tumor necrosis factor-α (TNF-α)[148], interleukin-1β (IL-1β), and interleukin-6 (IL-6)[149], can also lead to increased expression of EPO and the EPOR[80,95], and may provide a feedback loop that is regulated by EPO, such as TNF-α[150] (Fig. 1).

## FORKHEAD TRANSCRIPTION FACTORS OF THE "O" CLASS

### Background and Structure for FoxOs

Mammalian forkhead transcription factors of the O class (FoxOs) function to either block or activate target gene expression[83]. These proteins must bind to DNA through the forkhead domain that relies on 14 protein-DNA contacts. The forkhead domain in Fox proteins consists of three α-helices, three β-sheets, and two loops that are referred to as the wings[151], but not all winged helix domains are considered to be Fox proteins[152]. The forkhead domain is described as a "winged helix" as a result of a butterfly-like appearance on X-ray crystallography[151] or nuclear magnetic resonance imaging[153]. High sequence homology is present in the α-helices and β-sheets, with variations described in either absent β-sheets and loops or additional α-helices. Although both the first and second loops make contact with DNA, it is the second loop that can influence the stability of DNA binding. In addition, post-translational modification of FoxO proteins, such as phosphorylation or acetylation that block FoxO activity, alter the binding of the C-terminal basic region to DNA to prevent transcriptional activity[154]. Yet, other mechanisms may influence DNA binding of forkhead proteins, such as variations in the N-terminal region of the DNA recognition helix, changes in electrostatic distribution, and the ability of forkhead proteins to be shuttled to the cell nucleus[81,155].

In regards to the forkhead family, at least 100 forkhead genes and 19 human subgroups that range from *FOXA* to *FOXS* are now known to exist since the initial discovery of the fly *Drosophila melanogaster gene forkhead*[156]. The original nomenclature for these proteins, such as forkhead in rhabdomyosarcoma (*FKHR*), the *Drosophila* gene fork head (*fkh*), and Forkhead RElated ACtivator (FREAC)-1 and -2, has been replaced. The current nomenclature for human Fox proteins places all letters in uppercase, otherwise only the initial letter is listed as uppercase for the mouse, and for all other chordates, the initial and subclass letters are in uppercase[157]. FoxOs were first reported in fusion genes in human soft-tissue tumors and leukemias. FOXO1, termed forkhead in rhabdomyosarcoma (FKHR), and FOXO3a, also known as FKHRL1 (forkhead in rhabdomyosarcoma like protein 1), and their genes were identified through chromosomal translocations in alveolar rhabdomyosarcoma tumors[158]. The acute leukemia fusion gene located in chromosome X (*AFX*), also known as the *FOXO4* gene, was demonstrated as a gene that fused to MLL transcription factor as a result of the *t(X; 11)* chromosomal translocation in acute lymphoblastic leukemia[159]. A fusion between FOXO2 and MLL also occurs in some cases of acute myeloid leukemia that may be identical to FOXO3a[160].

## Expression and Regulation of FoxO Proteins

FoxO proteins (FoxO1, FoxO3, FoxO4, and FoxO6) are present throughout the body and are expressed in tissues of the reproductive system of males and females, skeletal muscle, the cardiovascular system, lung, liver, pancreas, spleen, thymus, and the nervous system[81,82,83,143,161,162,163,164,165,166,167] (Fig. 2). Interestingly, FoxO proteins are not equally expressed in all tissues, suggesting that individual FoxO proteins may have specificity in regards to cellular function[166]. For example, FoxO6 expression is found in several regions of the brain that play a significant role in cognitive function and emotion, such as the hippocampus, the amygdala, and the nucleus accumbens[164]. In contrast, FoxO1 may be more suited for the control of motor function and memory formation, since the expression of this protein is primarily in the striatum and subregions of the hippocampus[164]. In addition, FoxO3 is more diffusely represented in the hippocampus, cortex, and cerebellum, suggesting a complementary role for this FoxO protein to control cognitive and motor function. FoxO expression can be variable in other tissues[83]. Although studies in mice have shown that the mRNA distribution of Foxo1, Foxo3a, and Foxo4 is similar in the embryo and adult[162], Foxo1 expression was highest in adipose tissue, Foxo3a expression was greatest in the liver, and Foxo4 expression was strongest in muscle[162]. Subsequent work in mice has described Foxo1 expression in all tissues with high levels in the ovaries[168]. Foxo3a also is expressed in all tissues and Foxo4 expression was considered to be more tissue specific in skeletal muscle[168].

Post-translational control of FoxO proteins employs pathways associated with ubiquitylation and acetylation[169,170]. IκB kinase (IKK) can phosphorylate and block the activity of FoxO proteins, such as FoxO3a[81,158]. This leads to the proteolysis of FoxO3a via the Ub-dependent proteasome pathway[81,158,171,172,173]. FoxO proteins are also acetylated by histone acetyltransferases that include p300, the CREB-binding protein (CBP), and the CBP-associated factor. In addition, FoxO proteins are deacetylated by histone deacetylases. These include Sirt1, a $NAD^+$-dependent deacetylase, and the mammalian ortholog of the silent information regulator 2 (Sir2) protein[81], that can control multiple processes such as cell injury, life span, and metabolism[174,175]. Acetylation of FoxO proteins provides another avenue for the control of these proteins. Once acetylated such as by CBP, FoxO proteins may translocate to the cell nucleus, but have diminished activity, since acetylation of lysine residues on FoxO proteins has been shown to limit the ability of FoxO proteins to bind to DNA[176]. Acetylation also can increase phosphorylation of FoxO proteins by the serine-threonine kinase protein kinase B (Akt)[176].

In addition to acetylation and ubiquitylation, post-translational modulation of FoxO proteins also involves pathways associated with phosphorylation[81,158,171,172,173]. Protein phosphorylation is a critical pathway in the scheme for protein regulation[177]. Akt is a primary mediator of phosphorylation of FoxO1, FoxO3a, and FoxO4 that can block activity of these proteins[158,178] (Fig. 1). Akt phosphorylation of FoxO proteins not only retains these transcription factors in the cytoplasm, but also leads to ubiquitination and degradation through the 26S proteasome[170,171]. The serum- and glucocorticoid-inducible protein kinase (Sgk), a member of a family of kinases termed AGC (protein kinase A/protein kinase G/protein kinase C) kinases that includes Akt, can also phosphorylate and retain FoxO3a in the cytoplasm[179]. Knowledge that Sgk and Akt can phosphorylate FoxO3a at different sites suggests other avenues to prevent more effectively apoptotic cell injury that may be mediated by FoxO3a activity. Yet, phosphorylation of FoxO proteins does not always lead to negative regulation. The protein kinase mammalian sterile 20-like kinase-1 can also phosphorylate FoxO proteins directly and lead to their activation[180]. The ability of sterile 20-like kinase-1 to activate FoxO proteins may be linked to c-Jun N-terminal kinase (JNK), since sterile 20-like kinase-1 can increase JNK activation[181].

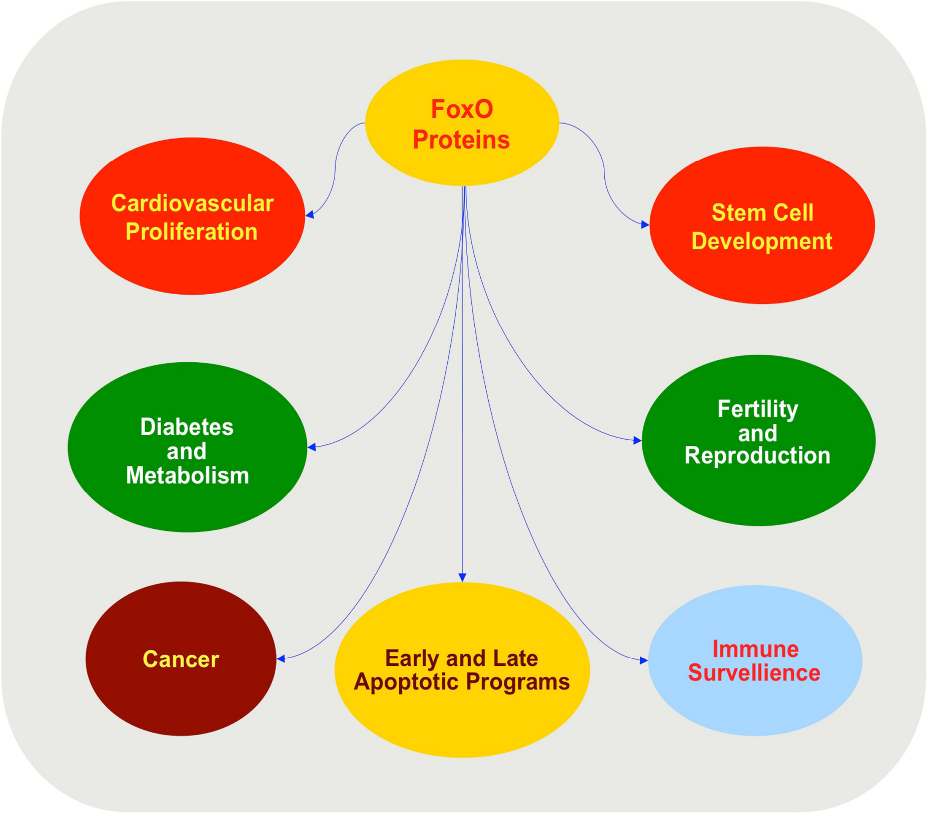

**FIGURE 2.** FoxO proteins govern a broad range of functions in the body. As transcription factors, FoxO proteins modulate multiple systems in the body. These include the initiation and development of stem cells, proliferation of the cardiovascular system, control of fertility and reproduction, modulation of metabolic pathways during homeostasis and disorders such as diabetes, immune system activation and surveillance, and the ultimate control of cell survival through early and late programs of apoptosis that can limit cancer progression.

Interestingly, activation of Akt in pathways that involve EPO or FoxOs is usually cytoprotective, but may mediate other processes. For example, Akt either alone or through EPO can lead to cell proliferation[182], blood-brain barrier permeability[183], cell protection during inflammation[184,185], neurodegeneration[186], hyperglycemia[187,188], hypoxia[112], Aβ toxicity[49,189,190,191,192], excitotoxicity[193], cardiomyopathy[194], cellular aging[195], and oxidative stress[30,32,42]. In addition, Akt can prevent cellular apoptosis through the phosphorylation of FoxO proteins[3]. Post-translational phosphorylation of FoxO proteins, such as during EPO administration, will maintain FoxO transcription factors in the cytoplasm by association with 14-3-3 proteins and prevent the transcription of proapoptotic target genes[80,113]. An exception to these observations involving the subcellular trafficking of FoxO proteins involves FoxO6. This FoxO protein usually resides in the nucleus of cells and is phosphorylated by Akt in the nucleus. FoxO6 does not contain a conserved C-terminal Akt motif, which limits nuclear shuttling of this protein, but FoxO6 transcriptional activity can be blocked by growth factors independent of shuttling to the cytosol through a FoxO6 N-terminal Akt site[196].

Modulation of Akt activity also controls apoptotic pathways of caspases that may offer an alternative mechanism to regulate FoxO proteins[82]. Caspases are a family of cysteine proteases that are synthesized as inactive zymogens that are proteolytically cleaved into subunits at the onset of apoptosis[44,197,198]. The caspases 1 and 3 have been linked to the apoptotic pathways of genomic DNA cleavage, cellular membrane PS exposure, and activation of inflammatory cells[46,60,67] (Fig. 1). Caspase pathways may be tied to the forkhead transcription factor FoxO3a, since increased activity of

FoxO3a can result in cytochrome c release and caspase-induced apoptotic death[113,199,200,201]. Pathways that can inhibit caspase 3 appear to offer a unique regulatory mechanism. For example, studies suggest that cell death pathways that rely on FoxO3a also appear to involve caspase 3 activation[50]. FoxO3a activity promotes caspase-induced apoptotic death[113,199,200,201], but inhibition of caspase 3 can also maintain the phosphorylated "inactive" state of FoxO3a to prevent cell injury[113,199,200]. Other work has shown that caspase 3 activity and cleavage is promoted during transfection of a triple mutant FoxO3a expression in which three phosphorylation sites have been altered to prevent inactivation of FoxO3a[202]. Furthermore, FoxO3a may control early activation and subsequent apoptotic injury in microglia during Aβ exposure through caspase 3[50]. Since Aβ exposure can facilitate the cellular trafficking of FoxO3a from the cytoplasm to the cell nucleus to lead potentially to "proapoptotic" programs by this transcription factor[50], one program in particular that may be vital for apoptotic injury appears to involve the activation of caspase 3. Aβ exposure leads to a rapid and significant increase in caspase 3 activity within 6 h following Aβ administration, but this induction of caspase 3 activity by Aβ requires FoxO3a, since loss of FoxO3a through gene silencing prevents the induction of caspase 3 activity by Aβ.

## EPO, FOXOS, AND CELLULAR METABOLISM

Both EPO and FoxOs play a significant role during cellular metabolism and metabolic disorders such as diabetes mellitus (DM). DM is a significant health concern for both younger and older populations[203,204]. Almost 18–20 million individuals in the U.S. and more than 165 million individuals worldwide suffer from DM. By the year 2030, it is predicted that more than 360 million individuals will be afflicted with DM and its debilitating conditions. Type 2 DM represents at least 80% of all diabetics, and is dramatically increasing in incidence as a result of changes in human behavior and increased body mass index[203,205]. Type 1 insulin-dependent DM is present in 5–10% of all diabetics, but is increasing in adolescent minority groups[203,205]. Furthermore, the incidence of undiagnosed diabetes and impaired glucose tolerance in the population raises additional concerns.

Patients with DM can develop immune dysfunction[206], cognitive disorders[206,207], hepatic dysfunction[208], renal disease[209], hematological disease[210], neurodegenerative disorders[2,143,205], and cardiovascular disease[205,211]. Interestingly, the development of insulin resistance and the complications of DM can be the result of cellular oxidative stress[203,205]. Hyperglycemia can lead to increased production of ROS in ECs, liver cells, and pancreatic β-cells[203,204,205]. Recent clinical correlates support these experimental studies to show that elevated levels of ceruloplasmin are suggestive of increased ROS[203,204,205]. Furthermore, acute glucose swings in addition to chronic hyperglycemia can trigger oxidative stress mechanisms, illustrating the importance for therapeutic interventions during acute and sustained hyperglycemic episodes[203,205].

In regards to EPO during DM, plasma EPO is often low in diabetic patients with anemia[212] or without anemia[213]. The inability of these individuals to produce EPO in response to a declining hemoglobin level suggests an impaired EPO response in diabetic patients[214]. However, increased EPO secretion during diabetic pregnancies may represent the body's attempt at endogenous protection against the complications of DM[215]. Similar to the potential protective role of insulin[216], EPO administration has been shown both in diabetics as well as nondiabetics with severe, resistant, congestive heart failure to decrease fatigue, increase left ventricular ejection fraction, and significantly decrease the number of hospitalization days[217]. *In vitro* studies with vascular cells exposed to elevated glucose have also demonstrated that EPO can significantly improve EC survival in a 1.0-ng/ml range[218]. EPO administration in patients can also significantly increase plasma levels of EPO well above this range of 1.0 ng/ml that has been associated with potential EPO cellular protection in patients with cardiac or renal disease[219,220], suggesting that the effects of EPO observed during *in vitro* studies may parallel the cellular processes altered by EPO in patients with DM[124]. Furthermore, EPO during elevated glucose and similar to other models of oxidative stress can block neuronal degeneration[221] and apoptotic DNA degradation in ECs in cardiac and vascular cell models[67,112,113,115,222]. Protection by EPO also is related to the maintenance of mitochondrial membrane potential ($\Delta\Psi_m$). Loss of $\Delta\Psi_m$ through the opening of the mitochondrial permeability transition pore represents a significant determinant for cell injury and the subsequent induction of apoptosis[28,69]. EPO has the capacity to prevent the depolarization of the mitochondrial membrane that also affects the release of cytochrome c[51,112,223].

Additional work suggests that proteins derived from the *Drosophila Wingless* (*Wg*) and the mouse *Int-1* genes may be associated with the complications of DM[36]. The Wnt proteins are secreted cysteine-rich glycosylated proteins that can control cell proliferation[224,225], differentiation, survival, and tumorigenesis[45,226]. These genes are present in several cellular populations[227], such as neurons, cardiomyocytes, ECs, cancer cells, and preadipocytes[2]. Abnormalities in the Wnt pathway, such as with transcription factor 7-like 2 gene, may impart increased risk for type 2 DM in some populations[228,229,230] as well as have increased association with obesity[231]. Yet, intact Wnt family members may offer glucose tolerance and increased insulin sensitivity[232] as well as protect glomerular mesangial cells from elevated glucose-induced apoptosis[233]. These observations suggest a potential protective cellular mechanism for EPO through Wnt signaling. Cell culture studies demonstrate that the Wnt1 protein is necessary and sufficient to impart cellular protection during elevated glucose exposure[218]. EPO maintains the expression of Wnt1 during elevated glucose exposure and prevents loss of Wnt1 expression that would occur in the absence of EPO during elevated glucose. In addition, blockade of Wnt1 with a Wnt1 antibody can neutralize the protective capacity of EPO, illustrating that Wnt1 is a critical component in the cytoprotection of EPO during elevated glucose exposure[218].

Metabolic signaling with FoxOs is conserved among multiple species including *Caenorhabditis elegans, D. melanogaster,* and mammals (Fig. 2). FoxO proteins are homologous to the transcription factor DAuer Formation-16 (DAF-16) in the worm *C. elegans* that can determine metabolic insulin signaling and lead to life span extension[234,235], suggesting a significant role for FoxO proteins in relation to mammalian cell function[81,158]. FoxO proteins can stimulate the insulin-like growth factor binding protein-1 (IGFBP1) promoter by binding to the insulin-responsive sequence (IRS)[236]. Both insulin and insulin-like growth factor-1 (IGF-1) can suppress this activity through activation of Akt[236,237].

Analysis of the genetic variance in *FOXO1a* and *FOXO3a* on metabolic profiles, age-related diseases, fertility, fecundity, and mortality in patients has observed higher $HbA_{1c}$ levels and increased mortality risk associated with specific haplotypes of *FOXO1a*[238]. These clinical observations may coincide with the demonstration in human endothelial progenitor cells that elevated glucose levels can reduce post-translational phosphorylation of FOXO1, FOXO3a, and FOXO4, and allow for the nuclear translocation of these proteins to initiate an apoptotic program in endothelial progenitor cells[239]. In experimental models, FoxO proteins may prevent the toxic effects of high serum glucose levels. Interferon-γ–driven expression of tryptophan catabolism by cytotoxic T-lymphocyte antigen 4 may activate Foxo3a to protect dendritic cells from injury in nonobese diabetic mice[240]. Additional studies demonstrated that adipose tissue-specific expression of Foxo1 in mice improved glucose tolerance and sensitivity to insulin during an elevated fat diet[241]. FoxO proteins may also protect against diminished mitochondrial energy levels known to occur during insulin resistance, such as in the elderly populations[203,204205]. In caloric-restricted mice that have decreased energy reserves, Foxo1, Foxo3a, and Foxo4 mRNA levels were noted to increase progressively over a 2-year course[163]. These observations complement studies in *Drosophila* and mammalian cells that demonstrate an increase in insulin signaling to regulate cellular metabolism during the up-regulation of FoxO1 expression[242].

It should be noted that the ability for FoxO proteins to maintain proper physiologic controls over cellular metabolism may be limited and occur only during specific circumstances. For example, mice with a constitutively active Foxo1 transgene have increased microsomal triglyceride transfer protein and elevated plasma triglyceride levels[243]. Studies in cardiomyocytes also suggest detrimental results with enhanced FoxO activity. Increased transcriptional activity of FoxO1, such as by the Sirt1 activator resveratrol, can diminish insulin-mediated glucose uptake and result in insulin resistance[244].

Overexpression of Foxo1 in skeletal muscles of mice also can lead to reduced skeletal muscle mass and poor glycemic control[245], illustrating that activation of FoxO proteins may also impair cellular energy reserves. Other studies that block the expression of Foxo1 in normal and cachectic mice[246], or reduce FoxO3 expression[247], show the reverse, with an increase in skeletal muscle mass or resistance to muscle atrophy. These results become especially relevant in patients with cancer and cachexia, since FoxO protein expression may further muscle wasting for these individuals. With this in mind, one potential agent to consider for the maintenance of cellular metabolism in patients is nicotinamide[11,44,248], an agent that can also inhibit FoxO protein activity[200]. In patients with DM, oral nicotinamide protects β-cell function, prevents clinical disease in islet-cell antibody-positive first-degree relatives of type-1 DM, and can reduce $HbA_{1c}$ levels[11,44,203]. Nicotinamide, which is closely linked to cell longevity pathways[249,250], may derive its protective capacity through two separate mechanisms of post-translational modification of FoxO3a. Nicotinamide not only can maintain phosphorylation of FoxO3a and inhibit its activity, but can also preserve the integrity of the FoxO3a protein to block FoxO3a proteolysis that can yield proapoptotic amino-terminal fragments[200].

## EPO, FOXOS, STEM CELL PROLIFERATION, AND VASCULOGENESIS

The observation that EPO may promote tumor proliferation[84,251] and the initial identification of FoxO proteins in soft-tissue tumors and leukemias, neoplasms now believed to contain cancer stem cells for tumor self-renewal[252], suggests that EPO and FoxO proteins may be closely tied to stem cell proliferation and differentiation. In regards to cell development for EPO, it can promote angiogenesis[67,107,112]. EPO has both a mitogenic and chemotactic effect that can lead to matrix metalloproteinase-2 production, cell proliferation, and vessel formation in EC lines[80,95]. In cultured human and bovine ECs, EPO stimulates EC proliferation and fosters the migration of ECs[253]. In neonatal mesenteric microvascular ECs, EPO also leads to vasculogenesis[254]. Angiogenesis by EPO offers an additional level of cytoprotection in various cell systems. For example, in models of cerebral ischemia, EPO promotes factors for angiogenesis, such as Tie-2 and Angiopoietin-2, that may assist with the restoration of cerebral blood flow to preischemic levels[255]. EPO-controlled angiogenesis may also play a significant role during renal inflammation and prevention of allograft rejection[256]. In addition, EPO may promote the viability of transplanted marrow stromal cells and enhance capillary density during experimental cardiac ischemia[257]. Although EPO-induced angiogenesis may impart beneficial effects to ischemic cells of the nervous and cardiovascular systems for nutrient and oxygen supply, other scenarios that involve ocular neovascularization may also seek to block or limit angiogenesis by EPO to prevent disease progression[258]. In clinical studies, EPO serum levels are also significantly associated with the number and function of circulating endothelial progenitor cells, and EPO can stimulate postnatal neovascularization by increasing endothelial progenitor cell mobilization from the bone marrow[259]. Recently, EPO has been shown to increase the motility of human bone marrow multipotent stromal cells[260], suggesting that EPO may lead to increased cell viability during oxidative stress via progenitor cell recruitment[261,262,263]. Interestingly, the ability of EPO to foster eythroid progenitor cell development is dependent on the inhibition of FoxO3a activity[79,80], but may also require regulation of specific gene expression through an EPO-FoxO3a association to promote erythropoiesis in cultured cells[264]. In addition, a close association with EPO[80,117,265] may also be required to modulate FoxO protein activity, such as during erythroid progenitor cell development[78,79], further indicating that use of EPO in patients with combined anemia and cancer may have unexpected detrimental effects[79,80].

When one considers progenitor cell proliferation for FoxO proteins, either simultaneous deletion of *Foxo1*, *Foxo3a*, and *Foxo4,* or single deletion of *Foxo3a* in mice prevents the repopulation of hematopoietic stem cells and leads to apoptosis in these stem cell populations[266,267] (Fig. 2). In regards to the reproductive potential of an organism, deletion of the *FoxO3a* gene results in the depletion of oocytes and subsequent infertility[268]. Other work using a mouse model of FoxO3a overexpression in oocytes suggests that FoxO3a also may retard oocyte growth and follicular development, and leads to anovulation and luteinization of unruptured follicles[269]. In clinical studies, a small percentage of women who suffer from premature ovarian failure have mutations in *FOXO3a* and *FOXO1a*[270]. In neuronal populations, FoxOs also may prevent stem cell proliferation, since the proliferation of human neural progenitor cells appears to require the inhibitory phosphorylation of FOXO3a[271].

Similar to EPO, FoxO proteins also play a significant role to modulate new vessel growth that can impact on cardiovascular development. FoxO proteins are intimately involved in EC development and angiogenesis. For example, *Foxo3a*$^{-/-}$ and *Foxo4*$^{-/-}$ mice develop without incidence and are indistinguishable from control littermates. However, mice that are singly deficient in *Foxo1* die by embryonic day 11 and lack development of the vascular system[272]. Additional studies illustrate that EC colonies in *Foxo1*-deficient mice fail to respond to vascular endothelial growth factor in a manner similar to wild-type ECs[273], suggesting that FoxOs are necessary for the development of vascular cells as well as for the biological response to cellular mediators.

During cardiac development, FoxO proteins also appear to be necessary to modulate cardiomyocyte proliferation. Both FoxO1 and FoxO3 are expressed during embryonic through prenatal stages in the developing myocardium. The expression of these FoxO proteins is believed to regulate cardiomyocyte growth negatively, since overexpression of FoxO1 blocks cardiomyocyte proliferation, but expression of dominant-negative FoxO1 leads to enhanced cardiomyocyte growth[274]. These observations may provide clues into the roles of FoxO proteins during cardiac hypertrophy. Atrogin-1, a protein that can block cardiac hypertrophy, may rely on the up-regulation of Foxo1 and Foxo3a to disrupt cardiac hypertrophy, since mice lacking atrogin-1 are susceptible to cardiac hypertrophy and do not yield increased expression of Foxo1 and Foxo3a[275]. In regards to smooth muscle cell growth, gene transfer of FoxO3a can inhibit neointimal hyperplasia through the prevention of vascular smooth muscle growth[276]. However, not all FoxO proteins may exert an inhibitory effect on vascular smooth muscle cells. FoxO4 may inhibit smooth muscle cell differentiation through the repression of the transcriptional coactivator of smooth muscle genes myocardin[277], but other work suggests that FoxO4 can also increase matrix metalloproteinase-9 expression to promote vascular smooth muscle migration and foster neointimal hyperplasia[278].

In consideration of the ability of FoxO proteins to regulate vascular smooth muscle cell proliferation, these transcription factors may have a significant clinical role in regards to disorders that involve hypertension and cardiac failure. Vascular smooth muscle cells are vital for the regulation of vascular tone and systemic arterial blood pressure. High flow states in vessels can reduce FoxO1 activity, resulting in the potential proliferation of vascular smooth muscle cells, vascular neointimal hyperplasia, and subsequent pathological states, such as hypertension[279]. Furthermore, $\alpha$1-adrenergic agonists that increase systemic blood pressure can have the reverse effect and stimulate the expression of FoxO1 and its nuclear translocation that ultimately may lead to apoptotic EC injury[280]. More than moderate levels of vessel cyclic stretch that can occur during hypertension may lead to the phosphorylation and inhibition of Foxo1 and Foxo3a in smooth muscle cells to further contribute to pathological smooth muscle cell proliferation[281]. In human as well as murine models of cardiac failure, increased expression of Fox transcription factors, such as FoxO1a, have also been observed to suggest a potential association of FoxO proteins with imminent cardiac failure[282].

## EPO, FOXOS, CELL SURVIVAL, AND THE IMMUNE SYSTEM

During a number of scenarios, EPO and FoxO proteins directly govern cell survival. With EPO, it can prevent cell injury during hypoxia[46,112,283,284,285,286], excitotoxicity[287,288,289], parasitic disease[290,291,292], endotoxin shock[293,294], free radical exposure[51,67,288], cardiac disease[295,296], A$\beta$ toxicity[192,297,298], pancreatic disease[299], and pulmonary disease[300,301]. EPO also represents a potential option for the prevention of retinal degeneration or neovascularization[302,303,304,305], as well as glaucoma[306]. Systemic application of EPO can also improve functional outcome and reduce cell loss during spinal cord injury[307,308], traumatic cerebral edema[309], cortical trauma[310], and epileptic activity[114,311,312].

In contrast to EPO cytoprotection, FoxO transcription factors more often lead to apoptosis during oxidative stress[3] (Fig.2). For example, forkhead transcription factors such as FoxO1 and FoxO3a must be present for oxidative stress to result in apoptotic cell injury[313]. FoxO3a in conjunction with JNK has also been shown to modulate an apoptotic ligand activating a Fas-mediated death pathway in cultured motoneurons[314], to lead to apoptosis through tumor-necrosis-factor–related apoptosis-inducing ligand (TRAIL) and BH3-only proteins Noxa and Bim in neuroblastoma cells[201], and to promote proapoptotic activity of p53[315]. In addition, loss of FoxO expression during oxidative stress is protective to cells. Protein inhibition or gene knockdown of FoxO1 or FoxO3a can lead to reduction in ischemic infarct size in the brain[316]. Removal of FoxO expression can also mediate protection of metabotropic glutamate receptors during vascular injury[199], enhance pancreatic β-cell or neuronal survival through $NAD^+$ precursors during oxidative stress[200], and provide trophic factor protection with EPO[113] and neurotrophins[317].

Interestingly, FoxOs are associated with neurodegenerative pathways such as Alzheimer's disease. The National Institute on Aging estimates that almost 5 million people have Alzheimer's disease in the U.S. Furthermore, more than 24 million people suffer from AD, presenile dementia, and other disorders of cognitive loss worldwide. In Alzheimer's disease[318], Aβ is toxic to cells[49,192,319,320,321] and can lead to oxidative stress[1,23,24]. Aβ is also associated with the phosphorylation of FoxO1 and FoxO3a that can be blocked with ROS scavengers[322]. A common denominator in the pathways linked to Aβ toxicity involves Wnt signaling[49,323] and β-catenin. The canonical Wnt pathway[324,325] involves β-catenin[45,226] and ties FoxO proteins and Wnt signaling together[36]. β-Catenin may increase *FoxO* transcriptional activity and competitively limit β-catenin interaction with members of the lymphoid enhancer factor/T-cell factor family[326]. This may lead to cell injury, since β-catenin has been demonstrated to be necessary for protection against Aβ toxicity in neuronal cells[49]. However, not all conditions with FoxOs may lead to cell injury. Some studies suggest that the loss of FoxO1, FoxO3a, and FoxO4 protein expression may actually lead to an increase in free radical release that can be responsible for oxidative stress[267]. Furthermore, FoxO proteins may be protective during aging and exercise, since FoxO3a activity may enhance vascular smooth muscle antioxidant properties in aged animals and be beneficial to the cardiovascular system during physical exertion[327].

Given the significant roles that EPO and FoxOs play during cell survival, which is tightly linked to the immune system and allergic disorders[83,328], it may come as no surprise that these proteins are closely associated with modulation of the immune system, not only in the brain, but also throughout the body (Figs. 1 and 2). For example, in the brain, microglia lead to the phagocytic removal of both neurons and vascular cells[30,32,39]. During inflammation, microglial cells require the activation of intracellular cytoprotective pathways[31,40] in order to proliferate and remove injured cells[43,329]. Microglia can also form a barrier for the removal of foreign microorganisms from the central nervous system, and promote tissue repair during neuronal and vascular cell injury[31,330]. Yet, microglia may lead to cell injury through the generation of ROS[69,331] and through the production of cytokines[332,333].

EPO can reduce cytokine gene expression in ECs exposed to tumor necrosis factor[222], prevent ulcer progression in cases of scleroderma[334], modulate inflammation during experimental autoimmune encephalomyelitis[335], reduce inflammation in murine arthritis models[336], and block primary microglial activation and proliferation during oxidative stress[46,192] to prevent phagocytosis of injured cells through pathways that involve cellular membrane PS exposure, Akt[30], and the regulation of caspases[46,67,337]. EPO can directly inhibit several proinflammatory cytokines, such as IL-6, TNF-α, and monocyte chemoattractant protein 1[80,338], and reduce leukocyte inflammation[339]. EPO may also foster the preservation of microglial cells for neuronal and vascular restructuring by preventing apoptotic injury in microglia[40,340].

In general, forkhead transcription factors also have an important role in maintaining immune system function. The forkhead family member FoxP3 can control the development and function of thymicderived CD4(+)CD25(+) regulatory T cells (Treg) that impart autoimmunity. Loss of FoxP3 can result in autoimmune disorders[341]. Additional studies demonstrate the expression of FoxP3 in tumor cells, such as melanoma[342], as well as in Tregs that may significantly affect patient mortality, since the increased presence of Tregs in cancer patients combined with FoxP3 expression in tumors may impair antitumor autoimmune responses and lead to high mortality[343].

In regards to FoxO proteins, these transcription factors may also influence early apoptotic membrane PS externalization. The ability to regulate early apoptotic membrane PS exposure[46] and inflammatory cell activity[32] can ultimately affect cell survival, since activated immune cells can lead to the phagocytic removal of injured cells or tumor cells[39,63]. Recent work suggests a relationship between the regulation of immune system activity and the induction of apoptotic pathways that are dependent on FoxO proteins. Prevention of inflammatory activation and apoptosis in the nervous system, such as in systemic lupus erythematosus in animal models, may require the up-regulation of different Fox proteins, such as FoxJ1 and FoxO3a, that can block NF-κB activation and interferon-γ secretion[344]. FoxO proteins may also work in concert with Fas signaling to clear activated T cells following a decrease in cytokine stimulation in patients with autoimmune lymphoproliferative syndromes[345], suggesting that activation of specific FoxO proteins may be beneficial for autoimmune disorders, but may impair treatments designed to target tumor cells through immune mediated pathways. Furthermore, in mice deficient for *Foxo3a*, lymphoproliferation, organ inflammation of the salivary glands, lung, and kidney, and increased activity of helper T cells result, supporting an important role for FoxO3a in preventing T-cell hyperactivity[346]. FoxO3a also appears to be necessary for neutrophil activity, since *Foxo3a*-null mice are resistant to models of neutrophilic inflammation that involve immune complex-mediated inflammatory arthritis[347]. Patients with rheumatoid arthritis and osteoarthritis show phosphorylation of FOXO3a in T lymphocytes as well as FOXO1 and FOXO4 in synovial macrophages, suggesting that loss of functional FOXO family members may lead to inflammatory cell activation in these disorders[348]. *FOXO1* gene transcript levels also are down-regulated in peripheral blood mononuclear cells of patients with systemic lupus erythematosus and rheumatoid arthritis[349], illustrating a potential etiology through the loss of functional FOXO proteins for these disorders and possibly providing a biomarker of disease activity. Other studies show that FOXO1 protein controls L-selectin expression that can regulate human T-lymphocyte trafficking[350].

## THERAPEUTIC CONSIDERATIONS FOR CANCER

The potential for the initiation or progression of cancer during EPO administration supports investigations that can elucidate the downstream mechanisms of this growth factor and cytokine in order to avoid unwanted clinical outcomes. In particular, the close association that EPO holds with FoxO proteins suggests potential avenues to limit or block tumor cell proliferation. FoxO proteins can control tumor growth through the induction of apoptosis and the blockade of cell cycle progression (Fig. 2). For example, FoxO3a and FoxO4 can promote cell cycle arrest in mouse myoblastic cell lines through modulation of growth-arrest and DNA-damage-response protein 45[78,81]. Treatment of chronic myelogenous leukemia cell lines with the Bcr-Abl tyrosine kinase inhibitor imatinib requires FoxO3a activation to antagonize cell proliferation and promote apoptotic cell death through increased TRAIL production[351]. In addition, the transcription factor E2F-1, which controls the induction of the cell cycle, has been reported in cell lines to increase the endogenous expression of FoxO1 and FoxO3a to lead to cell cycle arrest[352]. In contrast, the loss of FoxO3a activity in association with c-Myc, p27, and nuclear factor-κB (NF-κB) can result in cell cycle induction and malignant transformation of mouse cells in the presence of oncogene activation[81,158]. Other work suggests that FoxO proteins utilize the p53 upstream regulator p19(Arf) through Myc to block cell cycle induction and lymphoma progression[353].

Studies with prostate cancer have shown that the tumor suppressor phosphatase and tensin homolog deleted on chromosome ten (PTEN) is mutated in approximately 80% of tumors with the loss of FOXO1 and FOXO3a activity. In cell cultures, overexpression of FoxO1 and FoxO3a in prostrate tumor cell lines also leads to apoptosis, suggesting that FoxO1 and FoxO3a are necessary for limiting prostate cell tumor growth[167]. Inhibition of FoxO3a activity can result in enhanced prostate tumor cell growth[354], while agents that increase FoxO3a activity in both androgen-sensitive and androgen-insensitive prostate cell lines prevent prostate cancer cell progression[355]. Therapeutic strategies that rely on the overexpression of a nonphosphorylatable form of FoxO3a that cannot be inactivated can also sensitize prostate cancer cells to androgen withdrawal–induced apoptosis[356]. However, in prostate cell lines, FoxO3a can be a positive regulator of androgen receptor expression and therefore may play a complex role in prostate cancer cell proliferation and growth inhibition[357]. Other factors that control FoxO protein function may also play a role during prostate tumor progression. In prostate cancer cells, cyclin-dependent kinase 1 (CDK1) can become overexpressed and subsequently phosphorylate FOXO1 to block its transcriptional activity and contribute to prostate tumorigenesis[358]. In a similar manner, it has been shown that astrocyte-elevated gene-1 (AEG-1) can be up-regulated in clinical prostate cancer[359], possibly leading to activation of Akt that suppresses FOXO3a[360] and apoptosis in prostate tumor cells.

Initial investigations of FOXO3a in clinical breast cancer suggested that activation of FOXO3a was associated with lymph nodal metastasis and a poor prognosis[361]. In contrast to these observations, other work has shown that FOXO3a was inactivated by IKK and that inactivation of FOXO3a was associated with a poor prognosis in breast cancer[362], suggesting that FOXO3a subcellular localization and pathways that enhance its activity could be used not only as a biomarker assay, but also as therapeutic targets. Other work in breast cancer cells demonstrated the tumor repressive ability of FoxOs by illustrating that increased activity of FoxO3a in association with JNK in breast cancer cell lines[363] or in association with cyclin-dependent kinase inhibitor p27 in isolated human breast cancer cells can prevent breast cancer growth[364]. In addition, FoxO proteins may be able to modulate estrogen function and indirectly block breast cancer growth. Overexpression of FoxO3a in breast cancer cell lines can decrease the expression of estrogen receptor–regulated genes and inhibits 17β-estradiol (E2)–dependent breast cancer growth[365].

FoxO proteins may also represent a viable option to control tumor progression in other tissues. FoxO proteins can function as redundant repressors of tumor growth. For example, somatic deletion in mice of *Foxo1*, *Foxo3a*, and *Foxo4* results in the growth of thymic lymphomas and hemangiomas[366]. Other work illustrates that FoxO3a activation in colon carcinoma cell lines prevents tumor proliferation through Myc target genes that involve the Mad/Mxd family of transcriptional repressors[367]. In addition, the loss of FoxO3a activity may participate in oncogenic transformation in B-chronic lymphocytic leukemia[368] and in the progression of chronic myelogenous leukemia cell lines[351]. Furthermore, studies suggest that some proteins, such as the Kaposi's sarcoma–associated herpes virus latent protein LANA2, may specifically block the transcriptional activity of FoxO3a to lead to tumor growth[369]. In cell models of endometrial cancer, presensitization of cells to block Akt activation and foster transcription activity of FoxO1 enhances the effect of chemotherapy to limit tumor growth[370].

## CONCLUSIONS AND CONSIDERATIONS

Both EPO and FoxO transcription factors hold great potential to yield new strategies for the treatment of neurovascular injury, immune mediated diseases, metabolic disease, and cancer-related disorders, as well as to offer the ability to follow disease onset and progression as biomarkers. In reference to EPO, U.S. annual sale revenues for EPO have recently been reported to approach 9 billion dollars[371] and over 100 trials with the National Institutes of Health website (www.clinicaltrials.gov) presently exist that are either recruiting or in preparation to examine the role of EPO in patients with a variety of disorders that include anemia, cancer, cardiac ischemia, or spinal cord trauma. Although some cardiac injury studies do not always demonstrate a benefit with EPO[372,373], early studies in patients with anemia or who are on chronic hemodialysis have suggested a direct cardiac benefit from EPO administration[374,375]. In addition, EPO administration can improve exercise tolerance either during cardiac or renal insufficiency in patients with anemia and congestive heart failure[128,376], and that may be dependent on improved pulmonary function[377]. Furthermore, a randomized, concealed, multicenter trial of 1460 patients who received 40,000 U of epoetin alfa up to a 3-week maximum following intensive care unit admission for trauma demonstrated a reduced mortality[378].

Yet, EPO is not well tolerated with comorbid conditions such as congestive heart failure, hypertension[379], and neoplasms[143]. Some studies suggest that elevated plasma levels of EPO independent of hemoglobin concentration can be associated with increased severity of disease in individuals with congestive heart failure[380] and that EPO may contribute to vascular stenosis with intima hyperplasia[381]. Adverse effects during treatment with EPO are not uncommon, such as an increased incidence of thrombotic vascular effects[378] or the use of EPO in cancer patients receiving chemotherapy that has been associated with nonfatal myocardial infarction, pyrexia, vomiting, shortness of breath, paresthesias, and upper respiratory tract infection[382]. In addition, both acute and long-term administration of EPO can significantly elevate mean arterial pressure that may place patients with hypertension at risk[383].

Cancer progression has been another significant concern raised with EPO administration[84,384]. EPO and its receptor can be found in tumor specimens, may block tumor cell apoptosis through Akt[385], enhance metastatic disease[386], and complicate radiotherapy by assisting with tumor angiogenesis[387]. The potential for EPO to lead to neoplastic growth is not well defined or understood at this time[388]. A number of competing factors must be considered and weighed that include the possible benefits of EPO administration in patients with cancer, the synergistic effects of EPO with chemotherapeutic modalities[389,390], the potential protection against chemotherapy tissue injury[391], and the treatment of cancer-related anemia.

Additional considerations for EPO exist other than those associated with EPO abuse and gene doping[392,393,394]. EPO has been correlated with the alteration of red cell membrane properties, leading to a cognitive decrement in rodent animal models[80,95,338]. Development of potentially detrimental side effects during EPO therapy, such as for cerebral ischemia with increased metabolic rate and blood viscosity[395], could also severely limit the use of EPO. As a result, alternate strategies have been suggested. New proposals examine the role of targeted bioavailability for EPO, such as in bone marrow stromal cells genetically engineered to secrete EPO[396] and controlled release of EPO from encapsulated cells[397,398]. The passage of EPO entry into the central nervous system continues to attract significant interest[399] as well as does the use of novel intranasal routes for EPO administration[286]. The development of derivations of EPO to reduce erythropoietic activity and the potential associated vascular complications[287] have also been put forth as new directions for treatment. Yet these lines of investigation are not without limitations, since chemical derivatives of EPO can become absent of clinical efficacy[80,95] as well as possibly lose the ability to promote sustainable cytoprotective effects, such as neurogenesis[400] and angiogenesis[255,256,258,401].

In contrast to the concerns of EPO to promote cancer, FoxO proteins offer the potential to target and prevent neoplastic progression. The ability of FoxO proteins to control cell cycle progression and promote apoptosis supports the premise that FoxOs may be an important component for new strategies directed against tumorigenesis. For example, the use of triple mutant FoxO1 or FoxO3a expression in which three phosphorylation sites have been altered to prevent inactivation of this protein has been proposed to block melanoma tumors[202] and endometrial cancer[402]. Other work also offers additional support for the use of FoxO proteins as biomarkers of cancer growth. Down-regulation of the phosphatidylinositol 3 kinase and Akt pathways have been associated with increased transcript levels for FOXO1a and FOXO3a in clinical prostate cancer samples and may indicate the onset of precancerous changes or the progression of ongoing tumor growth[403]. Although loss of Akt activity in prostate cancer cells can result in enhanced FoxO3a activity and subsequent apoptosis of tumor cells[359], it is conceivable that early stages of cancer may lead to reduced Akt activity with insufficient levels of active forkhead transcription factors to limit tumor progression. In addition, the early and persistent expression of phosphorylated FOXO1a in gastric tumors may not only indicate the onset of cancer, but may also suggest an improved prognosis for patients[404].

The known mutations in FoxO proteins that exist in several disease entities may provide novel insights for the treatment of other disorders. Future analysis in larger populations of patients with premature ovarian failure and diabetes could strengthen our understanding of the role of FoxO proteins in these disorders. In addition, targeting the activity of FoxO1, FoxO3a, or FoxO4 in cardiac and endothelial cells may prevent the onset of pathological cardiac hypertrophy and neointimal hyperplasia that may result in atherosclerosis. Recent studies also suggest that the utilization and combination of multiple biomarkers may improve risk assessment for patients suffering from cardiovascular disorders[405]. These studies illustrate that FoxO proteins may serve as biomarkers of disease activity, such as in individuals with imminent cardiac failure[282].

However, similar to studies with EPO, FoxO transcription factors may have complex and sometimes detrimental clinical outcomes. For example, FoxO protein inhibition of cell cycle progression may not consistently lead to apoptotic cell death. Some investigations suggest that during oxidative stress, FoxO3a activation in association with Sirt1 can lead to cell cycle arrest, but not result in apoptotic cell injury[406]. Furthermore, during hypoxic stress, forkhead transcription factors, such as FOXO3a, may potentiate antiapoptotic pathways in breast cancer cells to further tumor growth[407]. FoxO proteins also have been linked to potential chemotherapy drug resistance with increased expression of MDR1 (P-glycoprotein) that has been associated with chemotherapy drug resistance in breast cancer cells. FoxO1 can stimulate the transcriptional activity of MDR1 that may promote increased tolerance of tumor cells[408]. In addition, the common pathways shared between Wnt and forkhead proteins may lead to other outcomes that alter the ability to control tumor growth[45,409]. FoxO proteins may assist with β-catenin activation in the Wnt pathway and lead to tumor cell proliferation[226]. In the presence of Wnt deregulation and increased β-catenin activity, tumorigenesis may ensue, such as with the proliferation of medulloblastoma tumors[252]. Therefore, the role of FoxO protein involvement in several disorders may not be consistently known and may be influenced by multiple parameters, such as tissue characteristics, cellular metabolic state, and the age of an individual.

## ACKNOWLEDGMENTS

This research was supported by the following grants (KM): American Diabetes Association, American Heart Association (National), Bugher Foundation Award, Janssen Neuroscience Award, LEARN Foundation Award, MI Life Sciences Challenge Award, Nelson Foundation Award, NIH NIEHS (P30 ES06639), NIH NIA, and NIH NINDS.
